# The Impact of Feeding Cannabidiol (CBD) Containing Treats on Canine Response to a Noise-Induced Fear Response Test

**DOI:** 10.3389/fvets.2020.569565

**Published:** 2020-09-22

**Authors:** Elizabeth M. Morris, Susanna E. Kitts-Morgan, Dawn M. Spangler, Kyle R. McLeod, Joao H. C. Costa, David L. Harmon

**Affiliations:** ^1^Department of Animal and Food Sciences, University of Kentucky, Lexington, KY, United States; ^2^College of Veterinary Medicine, Lincoln Memorial University, Harrogate, TN, United States

**Keywords:** cannabidiol, cbd, canine, behavior, fear, fireworks

## Abstract

Interest is increasing regarding use of Cannabidiol (CBD) in companion animals due to anecdotal evidence of beneficial behavioral and health effects. The purpose of this investigation was to evaluate the influence of CBD on behavioral responses to fear-inducing stimuli in dogs. Sixteen dogs (18.1 ± 0.2 kg) were utilized in a replicated 4 × 4 Latin square design experiment with treatments arranged in a 2 × 2 factorial, consisting of control, 25 mg CBD, trazodone (100 mg for 10–20 kg BW, 200 mg for 20.1–40 kg BW), and the combination of CBD and trazodone. A fireworks model of noise-induced fear was used to assess CBD effectiveness after 7 d of supplementation. Each test lasted a total of 6 min and consisted of a 3 min environmental habituation phase with no noise and a 3 min noise phase with a fireworks track. Plasma was collected 1 h before, immediately after, and 1 h following testing for cortisol analysis. Behaviors in each 3 min block were video recorded, and heart rate (HR) sensors were fitted for collection of HR and HR variability parameters. Research personnel administering treats and analyzing behavioral data were blinded as to the treatments administered. Data were tested for normality using the UNIVARIATE procedure in SAS, then differences examined using the MIXED procedure with fixed effects of treatment, period, time, and treatment x time interaction. Inactivity duration and HR increased during the first minute of the fireworks track compared with 1 min prior (*P* < 0.001 and *P* = 0.011, respectively), indicating the fireworks model successfully generated a fear response. Trazodone lowered plasma cortisol (*P* < 0.001), which was unaffected by CBD (*P* = 0.104) or the combination with CBD (*P* = 0.238). Neither CBD nor trazodone affected the duration of inactivity (*P* = 0.918 and 0.329, respectively). Trazodone increased time spent with tail relaxed (*P* = 0.001). CBD tended to increase HR (*P* = 0.093) and decreased the peak of low- and high-frequency bands (LF and HF, *P* = 0.011 and 0.022, respectively). These results do not support an anxiolytic effect of CBD in dogs given 1.4 mg CBD/kg BW/d.

## Introduction

Noise aversion or reactivity is one of the most common fearful behaviors in dogs, with 40 to 50% of dogs demonstrating at least one fearful behavior in response to noise exposure (1, 2). There is, however, considerable variation in the behavioral responses to noise. Some dogs will reduce activity while others become hyperactive. Some behavioral changes, such as panting and hiding, are mild, while others, like destructiveness and self-trauma, are more extreme and potentially hazardous to the health and well-being of both dog and owner (3). Such extreme and detrimental; stress associated with fear reduces overall health and lifespan (4, 5).

Despite the prevalence of noise aversion behaviors in dogs, they frequently go untreated with less than one-third of dog owners reporting that they would seek advice for the treatment of noise aversion (1). Potential treatment regimens for various noise aversion behaviors include systematic desensitization with a CD-based training system and administration of medications or natural products (3). There are several commonly prescribed drugs for the treatment of canine behavior disorders associated with fear and anxiety, including benzodiazepines, selective serotonin reuptake inhibitors, and tricyclic anti-depressants (6, 7). However, some owners may be hesitant to administer such medications, whether due to the possibility of undesirable side effects, personal bias against drug use, or cost. This has led to increased interest in the use of natural extract products to alter fearful behaviors, like dog-appeasing pheromones or oral supplementations such as L-theanine, a tryptic hydrolysate of milk protein and fish hydrolysate (8–12). Additionally, there has been renewed interest in the use of cannabinoids, cannabidiol (CBD) in particular, to regulate anxiety disorders in both humans and companion animals (13).

Cannabidiol is one of over 90 cannabinoids produced by *Cannabis sativa* and has been proposed to exert several beneficial effects, including acting as an anti-inflammatory, immunomodulatory, and anxiolytic agent (14–16). But unlike Δ^9^-tetrahydrocannabinol (THC), the other major cannabinoid produced by *C. sativa* that is toxic to dogs, CBD does not produce psychoactive effects due to its low affinity for the CB1 receptor (17). The potential anxiolytic effects of CBD have been attributed to several mechanisms, including its activation of 5-HT_1A_ receptors and its ability to indirectly activate cannabinoid receptors by inhibiting the metabolism of the endocannabinoid anandamide (18, 19). This has produced great interest in using CBD as a potential alternative to conventional therapies to reduce anxiety. While there is considerable work examining its use as an anxiolytic in human and rodent models [reviewed extensively in (19)], this effect has yet to be examined in a canine model. But despite the lack of evidence, canine anxiety, and noise aversion are some of the most common reasons that pet owners seek information on and administer CBD to their pets (20).

As interest in, and use of, CBD in companion animals continues to increase, there is a critical need for research evaluating both the safety and effectiveness of CBD use for canine anxiety. Therefore, the objective of the current study was to evaluate the influence of CBD on behavioral responses to fear-inducing stimuli in dogs, with the underlying hypothesis was that CBD would reduce fearful and anxious responses. This hypothesis was tested using a fireworks model of noise-induced fear and anxiety in which the effectiveness of CBD was assessed by comparing CBD to both a positive and negative control and to the combination of CBD with the positive control. All treatments were expected to reduce fearful and anxious responses compared to the negative control.

## Materials and Methods

This study was reviewed and approved by the Lincoln Memorial University (LMU) institutional animal care and use committee (protocol number: 1811-RES) prior to the start of the study. All housing and husbandry received were in accordance with the Animal Welfare Act, the Guide for the Care and Use of Laboratory Animals (8th ed.), and all applicable LMU SOPs.

### Subjects and Housing

Twenty-four intact, adult dogs (12 male, 12 female; 1 to 5 years old; 17.7 ± 3.9 kg) of various mixed breeds, including Cur, Lab, Hound, Boxer, Shepherd, Dane, Schipperkee, Springer Spaniel, and Pit mixes were received from a local shelter for inclusion in this study. The shelter was asked to provide dogs weighing 16 ± 4 kg. Additionally, the shelter was informed and gave consent for the use of the dogs for research purposes prior to their arrival. Prior to beginning the experiment, each dog had a complete blood count (CBC), and serum chemistry analysis (IDEXX Laboratories, Inc., Westbrook, ME) performed, along with physical evaluation by a veterinarian and a fecal examination to rule out any underlying disease that might preclude enrollment. Dogs were excluded if they demonstrated serious behavioral issues, such as human aggression that would endanger research personnel, were severely emaciated, classified as a body condition score < 2 on a 5-point scale (where one is emaciated and five is obese), or if their initial evaluations revealed an underlying disease that required more than routine treatments (such as heartworm positive dogs). Two dogs were excluded from the experiment due to positive heartworm tests and 4 additional dogs were excluded due to other health or behavioral concerns. Dogs were individually housed in 1.2 × 1.8 m cages within one of two dog kennels at the LMU DeBusk Veterinary Teaching Center. Dogs were stratified by sex and evenly distributed between the two kennels.

### Diets and Treatments

Dogs were fed Purina Pro Plan EN Gastroenteric Dry Dog Food (Nestle Purina Inc., St. Louis, MO) to meet the daily metabolizable energy requirements of intact adult dogs at maintenance, calculated as (70 ^*^ BW^0.75^) ^*^ 1.8 and split into two meals fed at ~0,730 and 1,830 h each day. Dogs were weighed and body condition scored (5-point scale) weekly and diets adjusted accordingly. Treatments were arranged in a 2 × 2 factorial and consisted of (1) control (placebo treats), (2) 1.4 mg CBD/kg BW/d, (3) Trazodone + 0 mg CBD, and (4) 1.4 mg CBD/kg BW/d + Trazodone. Trazodone was dosed at 100 mg for dogs weighing 10.0–20.0 kg and at 200 mg for dogs weighing 20.1–40 kg as recommended by the veterinarian and based on previous work (21). Because trazodone does not require an extensive adaptation period, trazodone tablets were dosed via a Pill Pocket (Mars Petcare US, Franklin, TN) the evening prior and morning of the behavioral assessment.

The CBD was a constituent of a proprietary industrial hemp extract (AgTech Scientific, Paris, KY) that was incorporated into treats and administered in the form of two treats daily, with each treat containing half the daily dose. Groups not receiving CBD treatment received control treats (0 mg CBD). Both control and CBD treats were composed of the following ingredients: chicken, chicken liver, Asian carp, catfish, and in the case of the CBD treats, industrial hemp extract. Dosage of CBD was selected based on a preliminary palatability study that assessed increasing levels of CBD inclusion on food and treat consumption (unpublished). Treats were formulated to include CBD at a dose of 2 mg/kg BW/d based on an estimation that dogs would weigh an average of 16 kg. However, based on the mean weight of dogs included on the study, actual dosage of CBD was 1.4 mg/kg BW/d.

Treats were offered solely as a reward upon kennel re-entry following twice daily exercise at ~0,700 and 1,800 h each day. Trazodone tablets hidden in Pill Pockets were administered at ~1,830 h the evening before and 1,000 h the morning of each noise-induced fear response test. Empty Pill Pockets were administered to the control and CBD treatment groups on those days to ensure that research personnel administering the treats were blinded as to the treatments administered.

### Testing Room and Equipment

The testing room was an ~2.72 × 3.38 m isolation room located on the opposite side of the building relative to where dogs were housed. The room contained a wall-mounted table and cabinets, a set of closed metal kennels, and a cloth dog bed. The dogs could interact with these objects, but none obstructed the dogs from view of the cameras. Two cameras (Model BRC-Z700, Sony Co., New York, NY and Model B07DQPS3KY, QallExpress International, China) were secured on opposite sides of the room near the ceiling–~2 m from the floor—to ensure the dogs would be within sight at all times. Dogs were isolated in the testing room; handlers monitored the dogs from the adjacent room via the cameras and could not be seen by dogs. Two Bluetooth speakers (Bose Co., Framingham, MA) were placed on opposite sides of the room near the cameras to create a surround-sound effect during the noise tests. Between each dog's test, the room was cleaned with Rescue™ Concentrate (Virox Animal Health, Oakville, ON, Canada), an accelerated hydrogen peroxide-based disinfectant.

### Acclimation

After intake and entrance into the study, all dogs were adapted to their environment, diet, daily routine, and the testing room for 3 d (Table 1), in which the dogs spent 6 min in the testing room where behavior was monitored, but not scored. A baseline open field test followed the 3-d adaptation, where dogs were placed in the testing room, behavior was scored, but no noise track was played (described below). The next day, a 6-min baseline fireworks test was conducted (described below). Both the open field test and baseline fireworks test were used solely to select dogs for inclusion in the study. Dogs not exhibiting at least one behavioral change between the open field test and the fireworks test; behaviors such as cowering, shaking, vocalization, destructiveness, or tail tucking, were excluded from the study. Dogs included in the study spent 6 min in the test room every day throughout the experiment to eliminate the possibility of behavioral changes due to the novel environment of the test room. In order to acclimate dogs to the testing procedure, heart rate monitor bands were placed on the dogs for each days adaptation to the test room. Additionally, blood draws were simulated on non-testing days by restraining dogs and holding off cephalic and jugular veins prior to placing them in the testing room. The fireworks test was conducted on the last day of each 7-d period (Table 1).

**Table 1:** Schedule of events.

**Study day**	**Key event**
−7 to −6	Animal intake, physical exam, and bloodwork (CBC/serum chemistry)
−5 to −3	Acclimation to diet, daily routine, and testing room
−2	Open field test
−1	Baseline fireworks test
1 to 4	Start of treatment 1 (Squares 1–4 started on consecutive days)
7 to 10	Period 1 Fireworks Test, start of treatment 2 evening after test
14 to 17	Period 2 Fireworks Test, start of treatment 3 evening after test
21 to 24	Period 3 Fireworks Test, start of treatment 4 evening after test
28 to 31	Period 4 Fireworks Test

### Open Field and Fireworks Tests

A fireworks model of noise-induced fear and anxiety was utilized to assess the effectiveness of the treatments. All dogs received from the shelter (*n* = 24) received 1 open field test and 1 baseline fireworks test. All dogs included on the study (*n* = 16) also received 1 fireworks test per 7-d period (5 total fireworks tests), each lasting 6 min. During the open field test, the dogs were placed in the testing room and their behavior was recorded in two 3-min blocks where no fireworks track was played in order to assess baseline behavior of dogs in the testing room. During the fireworks tests, the first 3-min block was the same as the open field test where no noise was played (**Pre-Noise**), and the fireworks track was played over a stereo speaker system (mean) during the second 3-min block (**Noise**).

In previous work using this model, a thunderstorm track was utilized to test the noise-induced fear response in dogs (9, 22). However, a fireworks video (https://www.youtube.com/watch?v=5eLcHJLDlI8) was used because according to Blackwell et al. (1) a larger percentage of dogs respond to fireworks than to thunderstorms. This noise-induced fear response test used in this study was a modified version of the one developed and validated by Araujo et al. (22). They utilized a 9-min test that included “before,” “during,” and “after” thunderstorm time points. Because they saw no behavioral differences (i.e., near door duration, inactivity duration) between the “during” and “after” thunder time points, the test for this study was shortened to 6 min, ending immediately after the fireworks track (Noise time point) ended. This allowed for the immediate post-test blood sample collection to be obtained more quickly after the fireworks test. The mean of 90 dB was selected based on previous work (9, 22) that were both successful in generating a response using equal or lesser decibel thunderstorm track. Behaviors in each 3-min time block were recorded and analyzed as separate time points (Pre-Noise and Noise).

### Experimental Design

Sixteen dogs were included in this study (7 male, 9 female; 1 to 4 years old, mean BW 18.1 ± 0.2 kg). Dogs were selected based on their behavioral response to the baseline noise-induced fear test (described above), in which behaviors such as cowering, shaking, vocalization, destructiveness, and tucking tail upon the start of the fireworks track indicated the dog was reactive to noise. These behaviors were selected as they have been previously used to assess noise reactivity (9, 12, 23). Included dogs were then arranged in a replicated 4 × 4 Latin Square design experiment in which dogs within each square (4 dogs per square) were randomly assigned to receive one of the four treatments each week (Periods 1–4). Each square was tested on successive days for scheduling purposes. Dogs received each treatment for a 7-d period prior to each of the noise-induced fear response tests (Table 1).

On testing days, all experimental procedures started at 1,200 h. CBD treats were administered ~4 to 6 hours prior to the test, and the morning dose of trazodone was administered ~2 to 4 h prior to the test. Dogs received the test at the same time each week. No washout period was included between treatment periods. At the time of the completion of this study (July 2018), there was little literature available on the pharmacokinetics of oral CBD administration. Samara et al. (24) reported that the half-life of IV CBD administration was 6 to 9 h but had no estimate for an oral dose. For a similar dose of trazodone, Jay et al. (21) reported a mean half-life of elimination of 166 min. From these half-lives, it was decided that the 7-d treatment period would be sufficient to allow for elimination of previous treatments prior to the next test while also allowing for acclimation to the next treatment. Additionally, time constraints on the availability of the kennels in which the dogs were housed prevented the inclusion of washout periods.

Because of scheduling constraints, the test started as soon as the dogs entered the testing room on testing days. This did not allow for either HRV or behavior to return to normal after movement from kennel to testing room. To account for this, only data from the last minute of the Pre-Noise time point was utilized to represent the behavior and HRV of dogs during that time point, which served as a reference of their normal behavior prior to the fireworks track starting. Additionally, only the first minute of the Noise time point was utilized to represent the dogs' behavior and HRV during that time point in order to assess the dogs' initial reaction to the fireworks tract.

### Data Collection

Consumption of food and treats, consistency of stool, frequency of elimination, activity during exercise, mucus membrane color, and other indicators of general health status were monitored twice daily by research personnel. Evidence of any adverse event—defined as any symptom occurrence that would not be expected in normal dogs—was also monitored. However, no adverse events were observed in any dogs following the administration of CBD treats during this study.

On the day of each fireworks test, blood samples (5 mL) were collected via jugular or cephalic venipuncture 1 h prior to testing, immediately after testing (5–10 min after cessation of noise exposure), and again 1 h post testing for cortisol and CBD analysis. Blood samples were collected into EDTA plasma tubes, centrifuged at 1,645 × g, and stored at −80°C for later analysis. Plasma samples were analyzed in duplicate for cortisol using a commercial radioimmunoassay kit (MP Biomedicals, LLC, Solon, OH). The sensitivity reported for the radioimmunoassay was 1.7 ng/mL, and the intra- and inter-assay coefficients of variation were 5.3–8.9% and 7.5–9.3%, respectively.

Polar H10 (Polar Electro Inc., Bethpage, NY) heart rate sensors were used for the collection of heart rate (HR) and heart rate variability (HRV) parameters via Bluetooth connection to an iPhone app (Heart Rate Variability Logger, Marco Altini). Parameters measured are defined in Table 2. In general, HR will increase and HRV will decrease in response to stressful stimuli as a result of an increase in sympathetic nervous system activity (25). Thus, an effective treatment would be expected to decrease HR and increase HRV, indicating higher parasympathetic activity. Just prior to the open field and fireworks tests, the heart rate monitor bands were placed around the chest of the dogs immediately behind the front legs, with the rubberized surface placed ventrally immediately behind the left front leg. Electrode gel was applied liberally to the rubberized surface of the transmitter band to promote conductivity. Due to all dogs having short hair and the use of electrode gel, dogs did not have to be shaved to promote conductivity.

**Table 2:** Definition of heart rate (HR) and heart rate variability (HRV) parameters.

**Parameter**	**Definition**
HR	Heart rate, bpm
AVNN	Mean beat-to-beat intervals, ms
SDNN	Standard deviation of beat-to-beat intervals, ms
RMSSD	Square root of the mean squared difference of successive RRs or inter-beat intervals, ms
pNN50	Percentage of successive RR intervals that differ by more than 50 ms, %
LF	Peak frequency of the low-frequency band (0.04–0.15 Hz)
HF	Peak frequency of the high-frequency band (0.15–0.40 Hz)
LF/HF	Ratio of LF-to-HF

Two cameras mounted ~2 m from the floor on opposite corners of the testing room continuously recorded all video and audio data for each test. The duration of behaviors given in Table 3 were logged by a single trained observer who was blinded to treatments using The Observer XT software (Noldus Information Technology Inc., Leesburg, VA). Three of the dogs included on the study had docked tails, and as such had no data on tail posture. The behaviors assessed were selected based on behavioral measures used in previous work evaluating canine anxiety and fear (5, 23, 26). Based on these previous studies, duration of fearful behaviors such as panting, cowering, and tail tuck were expected to increase during the fireworks test. Thus, an effective treatment was expected to decrease the duration of such fearful behaviors. Behaviors in different behavioral categories (i.e., Movement vs. Tail Posture) were not mutually exclusive, whereas behaviors within a behavioral category were mutually exclusive.

**Table 3:** Ethogram of behaviors tracked by a single trained observer blinded to treatments using The Observer XT (Noldus Information Technology Inc., Leesburg, VA).

**Behavioral category**	**Behavior**	**Definition used**
Movement	Inactive	Standing still, sitting, or laying down
	Cowering	Sudden cessation of movement in response to a stimulus
	Pacing	Frantically moving back and forth, restlessness
	Destruction	Scratching or chewing at room furnishings
Eyes	Facing door	Eyes are focused on the door of the room
	Glancing around	Eyes are shifting back and forth, possibly looking for the source of a sound
	Other	Eyes are focused on something else in the room
Ears	Ears relaxed	Ears are held in natural position
	Ears erect	Ears raised in response to stimulus
	Ears moving	Ears moving back and forth
Tail posture	Tail relaxed	Tail is not rigid and is lower than the top of the body
	Tail stiff	Tail is rigid and horizontal
	Tail wagging	Tail is wagging back and forth
	Tail tucked	Tail is tucked between hind legs
Muzzle	Barking	Emitting a short, loud sound
	Whining	Emitting a long, high pitch sound, often repeated
	Panting	Mouth open wide with tongue protruding while breathing heavily
	Licking	Using the tongue on own body or another object
	Yawning	Opening the mouth wide and inhaling
	Biting	Using teeth on the door or object

### Statistical Analysis

The normality of data distribution was tested using the UNIVARIATE procedure in SAS (SAS Institute, Cary, NC) on the residual of the data. In instances where data did not meet normality assumptions, statistical analysis was performed on the natural logarithm transformation of the data. However, data were then back transformed for reporting purposes. The standard error of the back transformed data was calculated from the confidence limits of the transformed data as follows: SEM = (back-transformed upper limit–back-transformed lower limit)/3.92. The denominator relates to the Z-value of a 95% confidence interval (± 1.96). Cowering, pacing, destruction, tail wagging, tail tucked, and all muzzle behaviors could not be analyzed due to insufficient occurrences that prevented data from meeting normality assumptions. With the exception of HR, pNN50, and HF, parameters were not normally distributed and were analyzed using the natural logarithm of the data.

Blood cortisol was then analyzed using the MIXED procedure in SAS including the fixed effects of CBD, trazodone, period (Weeks 1–4), time (−60, 0, and 60 min), the interaction of CBD and trazodone, and the interaction of CBD by trazodone by time. Random effects included square and dog nested within square and repeated effect of time. All behavioral and HRV data from the 1-min immediately prior to (Pre-Noise) and the first min of the noise-induced fear response test (Noise) were also analyzed using the MIXED procedure in SAS including the fixed effects of CBD, trazodone, period (Weeks 1–4), time (Pre-Noise and Noise), and all accompanying interactions. Random effects included square and dog nested within square and repeated effect of time. Effects were considered significant when *P* ≤ 0.05 and considered a tendency when *P* ≤ 0.10.

## Results

### Blood Cortisol

**Figure 1:**
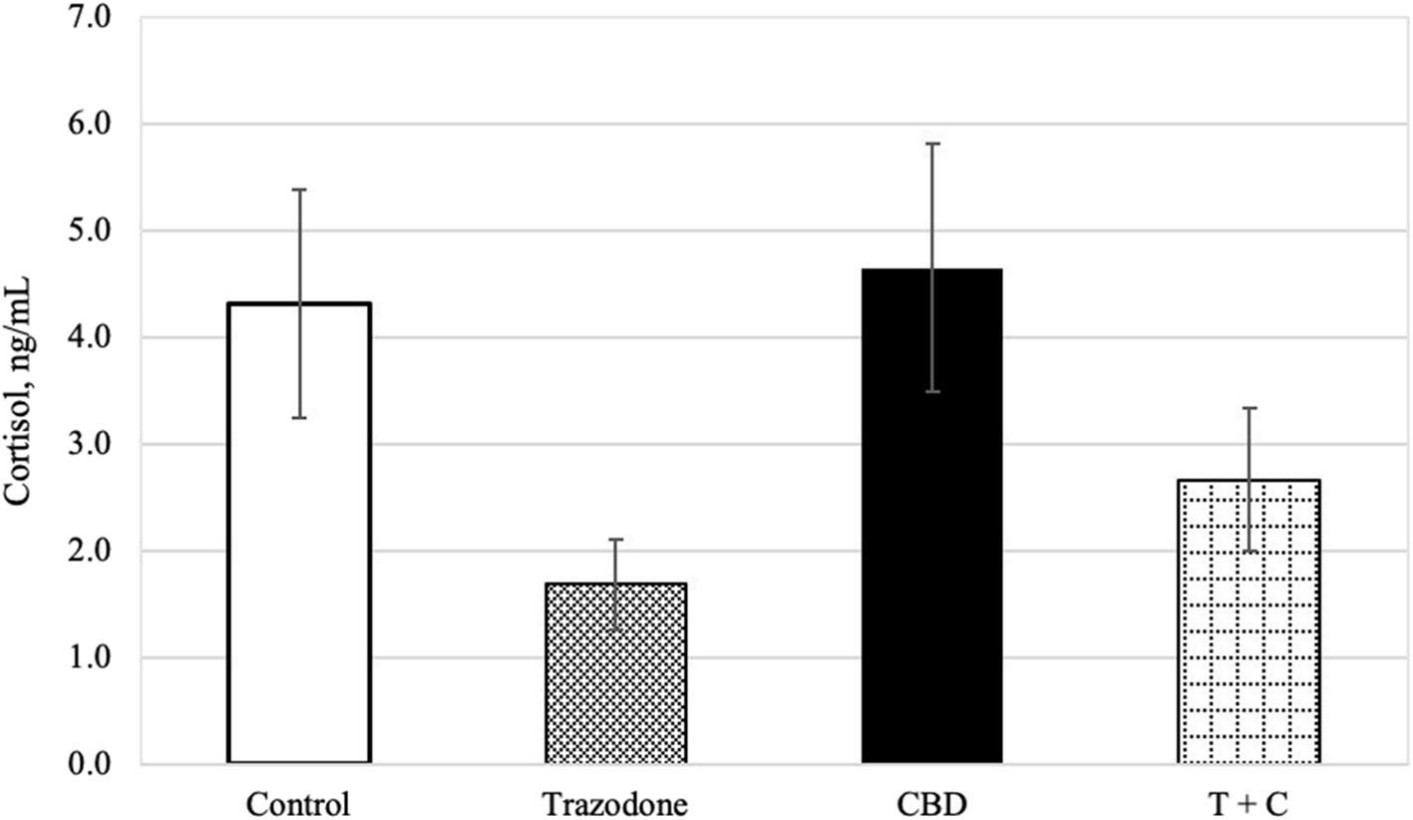
Cortisol concentration (ng/mL) for each treatment (*n* = 16), back transformed after analysis. Error bars represent the standard error of the treatment mean (SEM), which was calculated from the back-transformed confidence interval for each treatment: SEM = (upper limit—lower limit)/3.92. Due to lack of effect of time (*P* = 0.189) and any interactions with time (*P* > 0.05), all time points (Pre-Noise and Noise) have been combined. Trazodone treatment reduced cortisol concentration (*P* < 0.001), whereas there was no effect of CBD (*P* = 0.104) nor the CBD by trazodone interaction (*P* = 0.238).

There was an overall effect of period on blood cortisol (*P* = 0.024). Blood cortisol was reduced in period 1 compared to both periods 3 and 4 (*P* = 0.003 and 0.003, respectively), but was similar across all other periods (*P* > 0.05). Blood cortisol was unaffected by time of collection and CBD (*P* = 0.189, 0.104, respectively). Similarly, neither the CBD x trazodone, time × CBD, time × trazodone, nor the time × CBD × trazodone interactions affected blood cortisol (*P* = 0.238 0.772, 0.667, and 0.812, respectively). However, trazodone lowered blood cortisol concentrations (Figure 1; *P* < 0.0001).

### Heart Rate and Heart Rate Variability

There was a period effect on both HR and AVNN (Table 4; *P* = 0.005 and 0.046, respectively). Heart rate in period 4 tended to be lower than in period 1 (*P* = 0.075) and was lower than in periods 2 and 3 (*P* = 0.004 and 0.001, respectively). Heart rate was similar between periods 1, 2, and 3 (*P* > 0.05). The mean beat-to-beat intervals (AVNN) was increased in period 4 compared to all other periods (*P* = 0.021, 0.018, and 0.030, respectively), but was similar between all other periods (*P* > 0.05). All other HR and HRV variables were unaffected by period (*P* > 0.05).

**Table 4:** Effect of trazodone (T), CBD (C), CBD by trazodone (C^*^T) interaction, time (Pre-Noise and Noise), CBD by trazodone by time (C^*^T^*^Time) interaction, and period on mean heart rate (HR) and heart rate variability (HRV) parameters for 1-min immediately prior to (Pre-Noise) and the first minute (Noise) of the noise-induced fear response tests administered after each 7-d treatment period.

**Parameter**	**Treatment**				**SE^**1**^**	***P*****-value**					
	**Control**	**Trazodone (T)**	**CBD (C)**	**T+C**		**Trazodone**	**CBD**	C*T	**Time**	C*T*Time	**Period**
HR, bpm	118.03	118.02	124.20	124.07	10.968	0.985	0.093	0.987	<.001	0.637	0.005
AVNN, ms	555.96	539.85	539.42	517.50	25.988	0.276	0.266	0.850	0.040	0.807	0.046
SDNN, ms	108.16	102.96	106.54	88.18	7.129	0.200	0.359	0.450	0.977	0.419	0.695
RMSSD, ms	100.35	89.28	91.70	69.71	12.649	0.130	0.189	0.538	0.366	0.654	0.538
pNN50, %	41.19	36.99	36.88	33.73	5.774	0.180	0.168	0.847	0.032	0.773	0.306
LF, Hz	0.090	0.062	0.050	0.048	0.0071	0.188	0.011	0.315	0.010	0.273	0.533
HF, Hz	0.142^a^	0.076^b^	0.059^b^	0.068^b^	0.0221	0.205	0.022	0.071	0.036	0.853	0.481
LF/HF Ratio	0.729	0.803	0.711	0.540	0.1052	0.595	0.126	0.183	0.053	0.039	0.908
Pre-Noise	0.651^a^^,^ ^b^	0.992^a^^*^	0.907^a^^,^ ^b^	0.607^b^	0.1402						
Noise	0.808^a^	0.615^a^	0.515^a^	0.474^a^^*^	0.1461						

1 *The standard error (SE) of the back transformed data was calculated from the confidence limits of the transformed data as follows: SE = (back-transformed upper limit—back-transformed lower limit)/3.92*.

ab* *Within rows, values with different letters differ at P ≤ 0.05 and asterisks indicate a trend at P < 0.10*.

*Treatment T+C indicates the combination treatment of CBD and trazodone. With the exception of HR, pNN50, and HF, parameters were not normally distributed and were analyzed using the natural logarithm. Data were back transformed for reporting purposes. In the event of a treatment by time interaction, parameters are given as their treatment mean within each time point (Pre-Noise and Noise)*.

With the exception of SDNN and RMSSD, HR and all other HRV variables were affected by the time point (Pre-Noise vs. Noise) (Table 4; *P* < 0.05). HR was lower during the Pre-Noise time point compared to the Noise time point (*P* < 0.001), while HRV parameters affected by time—AVNN, pNN50, LF, and HF—were all higher during the Pre-Noise time point than the Noise time point (*P* < 0.05). The LF/HF ratio tended (*P* = 0.053) to be higher in the Pre-Noise time point compared to the Noise time point.

CBD tended to increase overall HR (Table 4; *P* = 0.093), and decreased LF regardless of time point (*P* = 0.011). All treatments reduced HF compared to control (*P* < 0.05). AVNN, SDNN, RMSSD, and pNN50 were unaltered by CBD and trazodone (*P* < 0.05). No HRV variables were affected by the CBD by time nor the trazodone by time interaction (*P* > 0.05). The CBD by trazodone by time interaction influenced the LF/HF ratio (*P* = 0.039). During the Pre-Noise time point, trazodone tended (*P* = 0.061) to increase the LF/HF ratio compared to control and increased the LF/HF ratio compared to the combination of CBD and trazodone (*P* = 0.038). During the Noise time point, the combination of CBD and trazodone tended (*P* = 0.083) to reduce the LF/HF ratio compared to control.

### Behavior

There were no period effects on any behavioral variables (Table 5; *P* > 0.05). With the exception of Facing Door and Tail Relaxed, all other behaviors were affected by time point (Pre-Noise vs. Noise; *P* < 0.05). During the Noise time point, duration of inactivity (*P* = 0.011), Glancing Around (*P* < 0.001), and Ears Moving (*P* < 0.001) were increased compared to their duration during the Pre-Noise time point. Conversely, the duration of Other Eyes, Ears Relaxed, Ears Erect, and Tail Stiff were reduced during the Noise time point compared to the Pre-Noise time point (*P* < 0.05). Across both time points, dogs fed CBD tended (*P* = 0.072) to spend less time focused on something in the room (Other Eyes). Conversely, trazodone increased overall duration of Other Eyes (*P* = 0.044) and time spent with Tail Relaxed (*P* = 0.001), but CBD did not alter tail posture (*P* = 0.753). No behavioral variables were affected by the CBD by time nor the trazodone by time interaction (*P* > 0.05).

**Table 5:** Effect of trazodone (T), CBD (C), CBD by trazodone interaction (C*T), time (Pre-Noise and Noise), CBD by trazodone by time interaction (C*T*Time), and period on the duration of behavioral parameters (s) for 1-min immediately prior to (Pre-Noise) and the first minute (Noise) of the noise-induced fear response tests administered after each 7-d treatment period.

**Parameter, s**	**Treatment**				**SE^**1**^**	***P*****-value**					
	**Control**	**Trazodone (T)**	**CBD (C)**	**T+C**		**Trazodone**	**CBD**	C*T	**Time**	C*T*Time	**Period**
Inactive	55.35	56.33	55.21	56.26	1.214	0.329	0.918	0.971	0.011	0.092	0.993
Facing door	37.45	33.90	34.96	37.70	4.198	0.872	0.796	0.217	0.561	0.556	0.786
Glancing around	16.90	15.65	17.93	15.91	3.460	0.396	0.736	0.841	<.001	0.142	0.819
Other eyes	5.10	13.33	4.10	5.48	1.885	0.044	0.072	0.182	<.001	0.469	0.792
Ears relaxed	11.37	7.76	12.35	11.43	4.913	0.179	0.168	0.422	<.001	0.868	0.567
Ears erect	29.33	34.29	29.93	29.80	5.614	0.304	0.408	0.279	<.001	0.747	0.982
Ears moving	19.25	17.79	17.20	18.78	2.076	0.970	0.742	0.351	<.001	0.457	0.493
Tail relaxed	37.90	49.86	38.93	50.96	4.857	0.001	0.753	0.992	0.611	0.898	0.990
Tail stiff	18.45	5.55	16.39	6.65	4.582	0.002	0.887	0.644	0.010	0.757	0.896

*Treatment T+C indicates the combination treatment of CBD and trazodone*.

1 *The standard error (SE) of the back transformed data was calculated from the confidence limits of the transformed data as follows: SE = (back-transformed upper limit—back-transformed lower limit)/3.92*.

*Parameters were not normally distributed and were analyzed using the natural logarithm. Data were back transformed for reporting purposes*.

These changes between the Pre-Noise and Noise time points may indicate that the fireworks test generated the desired fearful behavioral response. However, the behaviors Glancing Around and Ears Moving could be considered a normal response to hearing a loud noise and may not necessarily indicate a fearful response to the noise. However, since the common fearful behaviors measured—cowering, pacing, vocalizations, etc.—could not be analyzed due to insufficient occurrences, it is difficult to determine if the fireworks test was severe enough to generate a fearful response.

## Discussion

Since the passage of the Agriculture Improvement Act of 2018, which removed industrial hemp from the Controlled Substances Act and removed CBD from the Schedule I drug list, the market for industrial hemp-derived CBD has been able to expand considerably (27). Just 1 year after the act passed, the market was estimated to be $1.2 billion and is expected to grow to over $10 billion by 2024 (28). Much of this growth can be attributed to public perception of the supposed health benefits of CBD, including analgesic, antioxidant, anti-inflammatory, and anti-anxiety effects. However, despite general public opinion that CBD is a safe and effective treatment for these conditions, the lack of scientific clarity on the safety, dosage, and efficacy of CBD makes it critical for continued research in both humans and companion animals.

The present study is one of the first to describe the effect of CBD on the fear and anxiety response of dogs. The fear-response test was developed and validated by Araujo et al. (22), in which dogs were placed in the test room for 9 min and a thunderstorm track was played from 3 to 6 min. A modified version of this test was used in the current study, with a fireworks track being used instead of a thunderstorm track as previous literature has shown a greater percentage of dogs to be fearful of fireworks than of thunderstorms (1). Additionally, because Araujo et al. (22) saw no behavioral differences during the “after thunder” time period, the test for this study was shortened to 6 min, ending immediately after the fireworks track ended. This allowed for the immediate post-test blood sample collection to be obtained within 10 min of the end of the fireworks test.

If cortisol concentrations had decreased with each subsequent period of the experiment, it would have been an indication that the dogs were adapting to the sound stimulus. While there was a period effect on cortisol, it was due to cortisol in periods 3 and 4 being increased compared to period 1. This may indicate a heightened response to the sound stimulus upon repeated exposure, which suggests that the dogs were being conditioned to be stressed in the testing room despite being placed in the room on non-testing days to avoid such conditioning. The potential for conditioned place aversion is a limitation of the crossover design used in this study. It may be beneficial in future work to either include washout periods or utilize a different design to reduce the number of tests administered to each dog to prevent this conditioning; however, the latter would require a larger sample size than is needed when using a Latin Square design.

The lack of a time effect on blood cortisol concentration was also unexpected. It is possible that cortisol concentrations did not change because the fireworks test may not have produced a sufficient change in fear or stress in these dogs. However, Landsberg et al. (9) demonstrated that the use of a thunderstorm noise-induced fear response test—also averaging 90 dB—resulted in a time-dependent change in blood cortisol, with higher concentrations 5 min post-test compared to 1 h pre- and post-test samples. This time effect was not replicated in this study. Instead, cortisol concentrations decreased at each subsequent timepoint, though not enough to produce an overall effect of time. Other studies have also demonstrated that blood and saliva cortisol concentrations peak between 5 and 20 min after noise exposure and begin to decline as early as 30 min post-exposure (23, 29, 30). For this study, while the blood sample taken immediately after the test was taken within this window, it is possible that cortisol levels had not yet peaked after noise exposure. It would be beneficial in future work to take additional blood samples throughout the first hour after noise exposure to better show cortisol changes after noise exposure. Alternatively, it is also possible that the lack of time effect on cortisol may have been due to elevated initial stress due to the use of shelter animals. Franzini de Souza et al. (23) demonstrated differences in endocrine and behavioral responses between laboratory and companion dogs in response to sound stimuli. While shelter animals were not represented in that study, it is possible that increased stress from the shelter environment, transport, and new environment could impact cortisol concentrations and warrants further investigation.

It is also possible that the time of testing influenced cortisol concentrations. Kolevská et al. (31) showed that dogs not undergoing an exercise regimen had the highest blood cortisol concentrations between 1,000 and 1,300 h and the lowest concentrations between 1,600 and 1,900 h. A similar pattern was seen in this experiment, with the highest cortisol concentrations at the 60-min pre-test sample, which would have been taken between 1,200 and 1,400 h, and the lowest concentrations at the 60-min post-test sample period, which would have been taken between 1,400 and 1,600 h. While blood cortisol concentrations in samples taken from 1,300 to 1,600 h were lower than those taken between 1,000 and 1,300 h (31), that effect was not seen in this study. This could indicate that the noise-induced fear response test did in fact affect blood cortisol concentrations, maintaining the elevated levels through the afternoon rather than the normal drop expected from the circadian rhythm of the hormone. These results warrant further investigation, and future work should consider administering the noise test earlier in the day to account for possible influence of the circadian rhythm of cortisol.

In humans, trazodone has been shown to decrease plasma cortisol concentrations compared with placebo and is commonly prescribed for the treatment of anxiety, depression, and to facilitate sleep (7, 32). While trazodone is not currently labeled for use in dogs, off-label use of trazodone is common for the treatment of anxiety disorders as well as to reduce the agitation and distress associated with post-surgery confinement and reduced exercise (33, 34). In this experiment, treatment with trazodone lowered blood cortisol concentrations compared to all other treatments. On the other hand, CBD did not alter plasma cortisol concentrations compared to control in this experiment. In humans, CBD administration has been shown to attenuate the cortisol decrease associated with the circadian rhythm of the hormone (35, 36). While other anxiolytic supplements seem to reduce anxiety in dogs at least in part by reducing the cortisol response to stressors (9), the results of this study may suggest that CBD does not exert an anti-anxiety effect by lowering blood cortisol concentrations. However, Hurd et al. (37) demonstrated a decrease in salivary cortisol when CBD was dosed to humans at ~5 and 10 mg/kg BW, which may indicate that the CBD dosage selected for this study (1.4 mg/kg BW) was too low to exert an effect on cortisol.

Another possibility is that CBD was administered too early in the day of the fireworks test. Recent work with other oral CBD products with similar dosages to this study demonstrated the time of maximum CBD concentration to be around 1.5 h after administration and the half-life of elimination to be between 1 and 4 h (16, 38, 39). However, at the time this study was completed (July 2018), these works on CBD pharmacokinetics had not yet been published, and earlier literature (24) reported much longer half-life for IV administration of CBD. This resulted in CBD treats being administered between 4 and 6 h prior to the test in this study. In the future, it may be necessary to administer treatments within 2 h of the noise test in order for CBD to have the greatest effect. This was accounted for in the administration of trazodone, as Jay et al. (21) reported that the same dose of oral trazodone had a mean half-life of 166 min in dogs.

Even if CBD was administered too early to exert an anxiolytic effect, CBD did appear to inhibit the ability of trazodone to lower blood cortisol in the combination treatment compared with trazodone alone. This observation may support previous work that shows CBD to be a potent inhibitor of the cytochrome P450 family of enzymes, which is responsible for the metabolism of trazodone to its active metabolite, m-chlorophenylpiperazine, in the liver (40, 41). Several studies have highlighted these potential CBD-drug interactions as well as the lack of information regarding CBD doses that can be deemed safe for use—whether administered alone or in combination with other medications (42–45). The potential interaction between CBD and trazodone demonstrated in this study lends support to these concerns. While there has been some work investigating specific CBD-drug interactions (46), it may be inadvisable to administer CBD concomitantly with other products or medications until these interactions are more fully elucidated.

In agreement with previous work in both dogs and other species, oral CBD administration in this experiment was well-tolerated. No gastrointestinal or constitutional adverse events were observed in dogs receiving CBD during this study. Additionally, food consumption and body weight remained consistent throughout the experiment. However, other studies evaluating the safety of oral CBD administration in dogs have reported the potential for adverse events, including lethargy, gastrointestinal issues such as vomiting or diarrhea, and hematological changes such as increases in liver enzymes (16, 39, 47, 48). However, aside from initial bloodwork evaluated upon animal intake from the shelter, hematological changes were not evaluated during this experiment. As increases in liver enzymes may be indicative of altered liver function, the potential effects of oral CBD administration on clinical chemistry parameters should be monitored in future work.

Heart rate variability has been used as a measure of stress and anxiety in a number of animal species, including dogs. In particular, considerable work has been done using HRV as an indicator of canine fear and anxiety in response to stressful stimuli, in which HRV generally decreases and HR increases when animals are under stress, indicating impaired parasympathetic function and autonomic nervous system dysregulation (49–51). The results of this study concur, showing increased HR and decreased HRV—AVNN, pNN50, LF, and HF—during the fireworks stimulus compared to the Pre-Noise time point when no sound was played. As AVNN represents the interval between heart beats, the decrease in AVNN was expected alongside the increase in HR during the fireworks stimulus. The pNN50 is thought to relate to parasympathetic activity and was also expected to decrease with increased stress from the fireworks stimulus (25, 52). The low frequency band (LF) mainly reflects baroreceptor activity in the heart while at rest, but can be generated by parasympathetic, sympathetic, or baroreceptor activity depending on the situation. Unlike other HRV parameters, the LF band is expected to increase with stress as an increase in baroreceptor activity would be expected to accompany a rise in blood pressure (25, 53). This was not replicated in this study, where LF actually decreased during the fireworks stimuli. The high frequency band (HF), or respiratory band, corresponds to heart rate variations related to the respiratory cycle. Unlike LF, HF only reflects parasympathetic activity, and lower HF is correlated with stress and anxiety (54, 55). Because LF can be influenced by both sympathetic and parasympathetic activity while HF is only produced by parasympathetic activity, the LF/HF ratio has been used as a way to estimate sympathetic vs. parasympathetic activity (56). An increased LF/HF ratio is thought to indicate higher sympathetic drive, which would be expected when exposed to stressful stimuli and has been demonstrated in dogs exposed to sound stimuli (23, 29, 57). In this study, however, the LF/HF ratio tended to be reduced during the fireworks track compared to the Pre-Noise time point. This, combined with the reduction in LF, may indicate that the fireworks track was not sufficient to cause a fearful or stress response.

Additionally, the fireworks tract did not alter SDNN nor RMSSD in this study. The standard deviation of interbeat-intervals (SDNN) measures how interbeat-intervals change over time and has been shown to be reduced by stress (25, 52). As such, SDNN is generally measured over a 24 h collection period, though short-term periods have also been used to evaluate short-term variability (58, 59). The RMSSD reflects beat-to-beat variance and is used to estimate vagal mediated changes in HRV, which reflects self-regulatory capacity (56). Reduced RMSSD has been associated with smoking, high LDL cholesterol, and work stress in humans and has been shown to be reduced in sound-sensitive dogs in response to sound exposure (29, 55). As some of the findings of this study concur with previous work and other results conflict with what was expected upon exposure to fireworks, it is possible that the fireworks test was not successful in generating the desired fearful response. However, some of this conflicting evidence may be a result of the ultra-short time frame used for recording HRV, particularly for some variables that are more commonly measured over longer time periods. Future work should consider recording HRV over longer time frames in order to better assess changes. Only HR and AVNN were affected by the period of the experiment, where HR was reduced in period 4 and AVNN was increased in period 4 compared to all other periods. This may suggest that the dogs were acclimating to the fireworks stimulus, a limitation to this study design. Future work should consider either washout periods or a study design that does not require multiple noise-induced fear response tests in order to avoid this problem.

To our knowledge, no work has been done to evaluate the effect of CBD or trazodone administration on HRV in dogs, though there is some evidence that CBD may improve HRV in healthy humans (60). Since an increase in stress and anxiety due to sound stimuli has been shown to increase HR, LF, and the LF/HF ratio while decreasing RMSSD, and HF (23, 29, 57), it was expected that both CBD and trazodone would attenuate these changes. In contrast to these expectations, both LF and HF were decreased by CBD in this study compared to control. Conversely, CBD tended to increase HR, while SDNN, RMSSD, and pNN50 were unaffected by treatment. While the reduction in LF would indicate that CBD attenuated the increase in cardiac sympathetic modulation, the increase in HR and decrease in HF suggest the opposite. Trazodone, again in contradiction to expectations, reduced overall HF in this study, tended to increase the LF/HF ratio during the Pre-Noise time point, and did not affect any other HRV parameters. The combination of CBD and trazodone also reduced HF compared to control and tended to reduce the LF/HF ratio compared to all other treatments during the Noise time point when the fireworks track was playing. The lack of effect on other HRV parameters such as SDNN and RMSSD may be due to the fireworks track not producing a change in these variables rather than a lack of treatment effect. These conflicting results warrant further investigation, particularly considering the lack of information available regarding the effects of both CBD and trazodone on HRV in dogs.

When the fireworks track started, there was a visible change in the demeanor of the dogs compared to both the open field test and the first 3-min block of the noise-induced fear response tests (Pre-Noise). While this may indicate that the fireworks track was able to generate the desired behavioral response, it is also possible that the change in behavior was a result of the dogs' interest in the noise rather than a fearful response. However, the considerable variability in the type of observed responses makes it difficult to elucidate whether the change was due to fear or if it was just a reflexive response. The predominant response was a decrease in activity, which may or may not have been accompanied by a variety of other behaviors, such as a tucked tail, shaking, or nervous vocalizations like whining. These fearful behaviors would have been a better representation of the behavior changes due to the fireworks test as they have been used to evaluate such changes in other work (9, 23, 29, 57). However, these behaviors occurred too infrequently in this study to allow for statistical analysis. This may be indicative of a lack of behavioral response to the fireworks test. However, as all dogs were selected for this experiment based on the presentation of one or more fearful behaviors during baseline testing, this may simply highlight the variation in behavioral responses to sound exposure. Other anxiolytic supplements and medications have been shown to increase activity or distance traveled using this model (9, 22); however, neither CBD nor trazodone treatment changed activity compared to control. This is particularly surprising for the treatment groups receiving trazodone, which has previously been shown to visibly reduce behaviors associated with a number of stressful situations (34, 61, 62). However, several of these studies relied on owner-completed surveys rather than objective data to assess effectiveness.

In contrast, CBD has been shown to reduce anxious behaviors in mouse, rat, and human models, but at this time there is little to no literature regarding its effect on canine behavior. In mouse and rat models, responses to threatening or unpleasant stimuli were assessed by several methods, including the elevated plus-maze, Vogel-conflict test, contextual fear conditioning, and elevated T maze (63–65). The use of these models has shown that intraperitoneal administration of CBD in doses ranging from 1 to 20 mg/kg produced anxiolytic effects with some responses being dose-dependent (18, 19). Though different models of anxiety were used in rodents, this may indicate that a higher dose is necessary to produce the desired behavioral changes associated with reduced stress and anxiety, particularly if dosed orally due to the considerable first-pass effect on CBD in the liver (24, 66). Future research should investigate the effect of higher dosage of CBD for dogs above the dose tested in this study. Another important consideration is the time of CBD administration prior to noise exposure. As previously mentioned, oral CBD has been shown to have a half-life of <4 h (16, 38, 39), but CBD treats in this experiment were dosed between 4 and 6 h of testing. Thus, it is possible that the dose used in this study would be sufficient to generate an anxiolytic effect if dosed closer to the fireworks test. Alternatively, CBD may need to be dosed for longer than 7 days in order to produce anxiolytic effects. Future investigation into these possibilities is warranted.

While there was no period effect on any behavioral variables, the lack of behavioral response to treatment could also have been due to acclimation of some of the animals to the firework track. While dogs were selected for inclusion into the study based on their reaction to the baseline noise-induced fear response test, it is possible that the weekly exposure to the stimulus diminished the reaction of some of the dogs during the later tests. This hypothesis is supported by the effect of period on other variables measured in this study, including plasma cortisol, HR, and AVNN. This highlights an important limitation of this study design, where time constraints prevented washout periods. To avoid this issue in future work, dogs could be blocked by their reaction to the baseline test and assigned to just one treatment for the duration of the study. This would eliminate the need for multiple firework tests and would allow baseline and treatment tests to be spaced out over time but would also require a much larger sample size. However, considering the high level of variability in behavioral responses to the fireworks test, it would be difficult to ensure even distribution of dogs even with blocking. If feasible, it would be ideal to utilize the crossover design with longer washout periods to minimize the potential for acclimation to the stressful stimulus. The variability in behavioral responses also makes it difficult to quantify different fear responses. Several of the most common fearful behaviors (shaking, cowering, panting, etc.) were measured, but could not be analyzed due to insufficient occurrences, which may be accounted for in future work by aggregating such behaviors together into one behavioral category. The inclusion of a non-fearful control group should also be considered for future work as it would allow for better evaluation of changes in fearful behaviors in reactive dogs.

## Conclusions

The results of the current study do not provide strong support of an anxiolytic effect of CBD in dogs when supplemented at 1.4 mg CBD/kg BW/d. Trazodone, but not CBD, decreased plasma cortisol concentration. When combined with trazodone, CBD appeared to attenuate the effects of trazodone on plasma cortisol. Cannabidiol decreased LF and HF, tended to increase HR, and tended to decrease duration of Other Eyes. Conversely, trazodone increased duration of Other Eyes, increased time spent with tail relaxed, reduced HF, increased the LF/HF ratio.

It would be beneficial in future studies to use increasing doses of CBD to clarify any potential anxiolytic effect, if present, and the dose necessary to elicit that effect. This study demonstrates the considerable variation in canine anxiety behaviors, which makes it difficult to accurately measure the response to treatments. It may be inadvisable to administer CBD concomitantly with other products or medications as the results from this study highlight potential drug interactions associated with CBD use. Considering the increased interest of CBD use in companion animals, continued research is essential to understanding the mechanisms by which CBD may exert anxiolytic effects as well as possible risks, like drug interactions, associated with CBD administration.

## Data Availability Statement

The raw data supporting the conclusions of this article will be made available by the authors, without undue reservation.

## Ethics Statement

The animal study was reviewed and approved by Lincoln Memorial University IACUC.

## Author Contributions

DH, SK-M, KM, and EM contributed to the conception and design of the study. EM, SK-M, and DS collected data and blood samples. JC provided behavior analysis software. EM performed sample and data analyses, statistical analysis, and wrote the first draft of the manuscript. All authors contributed to manuscript revision, read, and approved the submitted version.

## Conflict of Interest

The authors declare that the research was conducted in the absence of any commercial or financial relationships that could be construed as a potential conflict of interest.
